# Synthesis and Investigation of Antibacterial Properties of Thymol, Carvacrol, Eugenol, and Perillyl Alcohol Based β‐Halo Alcohol and β‐Halo Thiol Compounds

**DOI:** 10.1002/jbt.70171

**Published:** 2025-02-17

**Authors:** Fatma Çakmak, Hande Toptan, Hayriye Genc Bilgicli, Mehmet Köroğlu, Mustafa Zengin

**Affiliations:** ^1^ Chemistry Department Science Faculty, Sakarya University Sakarya Turkey; ^2^ Sakarya University Training and Research Hospital, Medical Microbiology Sakarya Turkey; ^3^ Clinical Microbiology Department Medicine Faculty, Sakarya University Sakarya Turkey

**Keywords:** β‐halo alcohol, β‐halo thiol, antibacterial agents, carvacrol, eugenol, perillyl alcohol, thymol

## Abstract

A total of 12 new β‐halo alcohols and 12 new β‐halo thiol derivatives were synthesized. Natural alcohol compounds with known pharmacological properties were selected as starting substrates, aiming to synthesize compounds that have the potential to exhibit biological activity. The synthesis of β‐halo alcohol derivatives involved a two‐step process, while β‐halo thiol derivatives were carried out in three steps. Effective and inexpensive methods were used for all transformations. Yields for β‐halo alcohol derivatives ranged from 79% to 82%, and for β‐halo thiol derivatives from 66% to 71%. Their antibacterial properties against some gram (+) (*Staphylococcus aureus*, *Enterococcus faecalis*) and gram (−) (*Escherichia coli, Klebsiella pneumoniae, Pseudomonas aeruginosa*) strains were investigated. The antibacterial effects of 24 newly synthesized compounds were compared to commercially available antibiotics Chloramphenicol and Streptomycin.

## Graphical abstract

Synthesis and investigation of antibacterial properties of some new β‐halo alcohol and β‐halo thiol compounds.

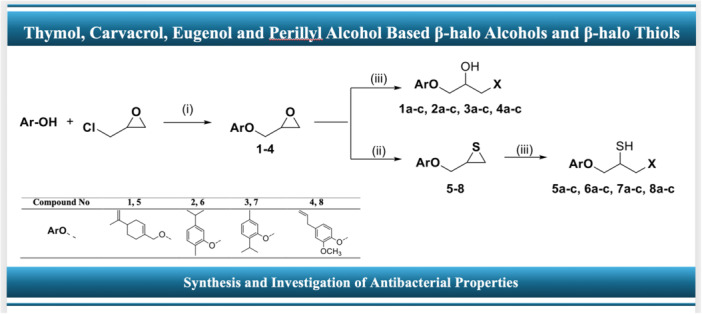

## 1 Introduction

Organohalogens are organic compounds containing at least one halogen atom (fluorine, chlorine, bromine, or iodine) in their structure. Over the past 40 years, the number of organohalogens discovered in nature has increased dramatically, from 200 to 3800. This surge is attributed mainly to renewed interest in natural products as potential sources of new medicinal drugs. Notably, marine ecosystems have proven to be a rich source of organohalogens, with discoveries spanning marine plants, animals, and bacteria. While many biogenic organohalogens are marine‐derived, they are also found in terrestrial plants, fungi, bacteria, and even higher organisms, including humans [1, 2, 3, 4, 5].

Halohydrins, or β‐halo alcohols, represent a specialized class of organohalogens characterized by a hydroxyl and halogen atom on adjacent carbons. These compounds, which can be synthesized via the addition of halogens to alkenes or epoxide ring opening [6, 7], are naturally produced by various organisms and exhibit diverse biological roles, including defense mechanisms [8, 9, 10]. Halohydrins can form in natural environments through interactions between halogens, water, and organic matter, particularly in marine settings [11, 12, 13, 14, 15, 16]. Marine organisms, such as algae and sponges, are notable producers of halohydrins [17, 18, 19].

**Figure 1:**
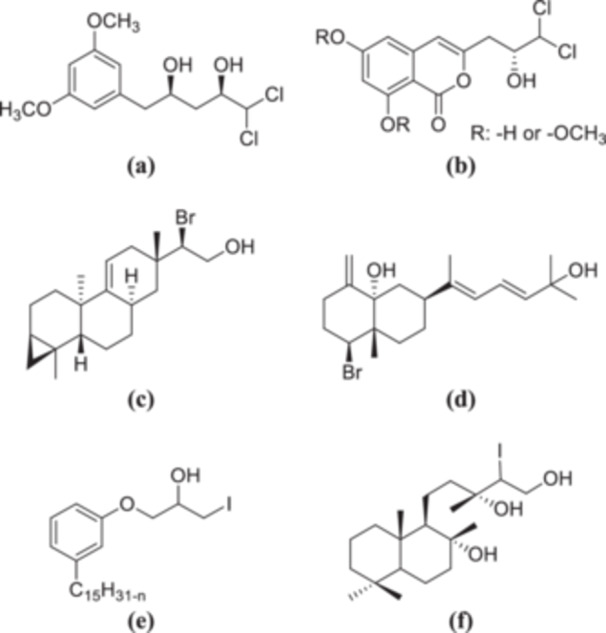
Samples of natural or synthetic origin containing hydroxide and halogen in their structure (a) citreochlorol, (b) halogenated metabolites in the secondary metabolism of *Aspergillus oryzae RIB40* (Koji mold), (c) eudesmane derivatives isolated from marine red alga *Laurencia pinnata*, (d) the red algae *Borneon Laurencia* spp. metabolites of the red algae *Borneon Laurencia* spp., (e) a synthetic iodohydrin derivative of cardanol, (f) the synthetic sclareol derivative.

Figure 1 shows examples of natural and synthetic compounds with hydroxyl and halogen in their structures. Citreochlorol, a naturally occurring polyketide, is isolated primarily from various fungal species (Figure 1a) [20]. Halogenated metabolites are also present in the secondary metabolism of *Aspergillus oryzae RIB40* (Koji mold), a fungus widely used for food fermentation in Asian countries (Figure 1b) [21]. Halogenated eudesmane derivatives have been isolated from the marine red alga *Laurencia pinnata* collected from Nanji Island, China (Figure 1c) [22]. Additionally, halogenated metabolites have been identified in red algae of the genus *Laurencia* spp. produce halogenated metabolites having strong antibacterial activity against clinical bacteria such as *Staphylococcus aureus* (*S. aureus*), *Staphylococcus* sp., *Streptococcus pyogenes, Salmonella* sp., and *Vibrio cholerae* (Figure 1d) [23]. A synthetic iodohydrin derivative of cardanol exhibits potential as a commercial drug to combat vector mosquito larvae with an LC50 of 0.0023 ppm after 72 h of exposure (Figure 1e) [24]. Additionally, a synthetic sclareol derivative has shown higher antifungal activity against certain phytopathogenic fungi than the natural product sclareol (Figure 1f) [25]. Despite these advancements, the biological potential of β‐halo thiols (1‐halo‐2‐thiol) remains underexplored. These compounds, primarily considered precursors in heterocyclic synthesis, may hold untapped pharmaceutical potential [26, 27].

Terpenoids, known for their roles in plant color, aroma, and development, also exhibit therapeutic properties, including antibacterial activity [28]. However, their clinical application is often limited by high volatility and short action duration [29, 30]. Addressing these challenges, the modification of natural compounds with known antibacterial properties is a key strategy in medicinal chemistry [31]. Moreover, based on our experience from previous studies, we have seen that certain pharmacological properties, such as antibacterial activity or enzyme inhibition, can be improved by derivatizing terpenoids [32, 33, 34, 35].

For this purpose, new β‐halo alcohols and β‐halo thiols were synthesized by selecting terpene derivatives from essential oil components, known for their antibacterial activity since ancient times. The antibacterial activities of the obtained new derivatives were investigated. For this purpose, the antibacterial properties of all compounds against gram (+) (*S. aureus*, *Enterococcus faecalis* (*E. faecalis*)) and gram (−) (*Escherichia coli* (*E. coli*), *Klebsiella pneumoniae* (*K. pneumoniae*), *Pseudomonas aeruginosa* (*P. aeruginosa*)) strains were investigated by microdilution method and compared with commercially available antibiotics Chloramphenicol and Streptomycin.

## 2 Results and Discussion

### 2.1 Chemistry

**Scheme 1:**
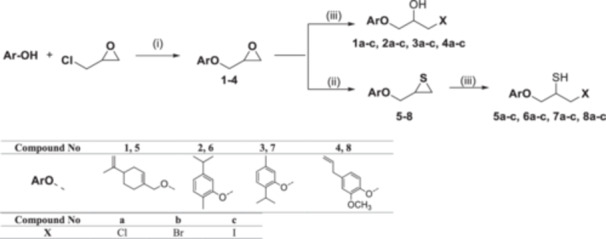
Reaction conditions (i) K_2_CO_3_, TBAB, rt for **1**; KOH, MeOH, 65°C for **2**, **3**, and **4**; (ii) Thiourea, MeOH, 65°C; (iii) LiX, AcOH, THF.

Four natural alcohol‐derived compounds, eugenol, thymol, carvacrol, and (*S*)‐perillyl alcohol, were selected as starting materials. In the first step, these alcohols were treated with epichlorohydrin in a basic medium to obtain oxirane derivatives. After that, halohydrin compounds were obtained by opening the oxirane ring with lithium halogen salts in acetic acid. On the other hand, oxirane derivatives were obtained by reacting them with thiourea in a methanol environment. Thirane derivatives were opened with lithium halogen salts to get 1‐halo‐2‐thiol compounds (Scheme 1). Structures of synthesized compounds and obtained total yields are given in Table 1.

**Table 1:** Structures of synthesized compounds and obtained total yields.

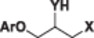		Ar: Eugenol, Timol, Carvacrol, (*S*)‐Perillyl alcohol *Y*H: OH, SH *X*: Cl, Br, I			
Compound	Yield (%)	Compound	Yield (%)	Compound	Yield (%)
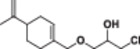	75	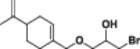	73	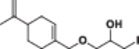	75
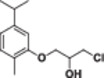	79	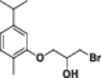	82	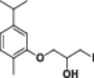	81
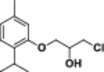	73	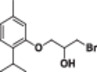	76	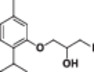	76
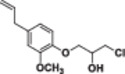	71	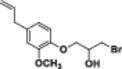	72	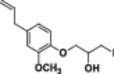	71
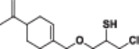	67		67	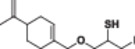	68
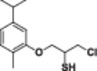	67	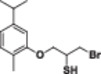	68	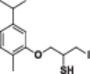	68
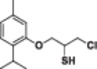	66	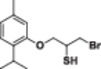	67	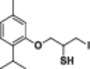	68
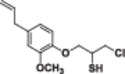	71	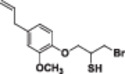	71	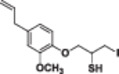	71

### 2.2 Antibacterial Studies

Over the past 20 years, the rise in antibiotic resistance and the limited development of new antibiotics have led to an increasing number of studies on the effectiveness of alternative antimicrobial substances [36]. The antimicrobial activities of oxypropanolamine compounds with added thymol, eugenol, and carvacrol are well‐documented [37]. This study aims to synthesize 12 new β‐halo alcohols and 12 new β‐halo thiols and examine their antimicrobial activities.

Among the β‐halo alcohol compounds, **2a** was found to have lower antibacterial activity than the others for *S. aureus*. For *E. faecalis*, **4a**, **4b**, and **4c**, which are all eugenol derivatives, were found to have lower activity than the other β‐halo alcohol derivatives. For *E. coli*, **1b** and **4a** were found to be less effective, and **2a**, **3a**, **3c**, and **4b** were found to be more effective. For *K. pneumoniae*, **4a** was found to be the least effective, and **1c** and **3b** were found to have higher antibacterial activity. For *P. aeruginosa*, **1b**, **2b**, **3b**, and **4b** were the most effective. Notably, oxirane derivatives of eugenol have lower antibacterial activity than other β‐halo alcohol derivatives against *E. faecalis*, and the most effective β‐halo alcohol derivatives against *P. aeruginosa* are always bromide‐added compounds. It would be appropriate to support the higher antibacterial activity of bromide compared to chloride or iodine for *P. aeruginosa* in further studies (Table 2).

**Table 2:** Minimum inhibitory concentrations (μg/mL) of compounds 1a–c, 2a–c, 3a–c, and 4a–c against some common gram‐positive and gram‐negative bacteria.

	1a	1b	1c	2a	2b	2c	3a	3b	3c	4a	4b	4c	Clor*	Str**
*S. aureus*	234.4	234.4	234.4	1875	234.4	234.4	234.4	234.4	234.4	234.4	234.4	234.4	16	8
*E. faecalis*	234.4	234.4	468.8	234.4	234.4	234.4	234.4	234.4	234.4	937.5	468.8	937.5	256	256
*E. coli*	3750	7500	3750	1875	3750	3750	1875	3750	1875	7500	1875	3750	16	16
*K. pneumoniae*	3750	3750	1875	3750	3750	3750	3750	1875	3750	7500	3750	3750	128	256
*P. aeruginosa*	1875	1875	3750	1875	1875	3750	7500	1875	3750	7500	1875	1875	512	64

* Chloramphenicol.

** Streptomycin.

When β‐halo thiol compounds were examined, the most effective antibacterial activity for *S. aureus* was **5c**, while the lowest antibacterial activity was **8b** and **8c**. The highest activity for *E. faecalis* was observed in **6a** and **7a,** and the lowest activity was observed in **8a**, **8b**, and **8c**. For all three gram‐negative bacteria studied (*E. coli*, *K. pneumoniae, P. aeruginosa*), regardless of species, compounds **8a**, **8b**, and **8c** showed lower antibacterial activity than the others. It was observed that all β‐halo thiol compounds that showed lower activity than others in gram‐negative bacteria and *E. faecalis* were eugenol based (Table 3).

**Table 3:** Minimum inhibitory concentrations (μg/mL) of compounds 5a–c, 6a–c, 7a–c, and 8a–c against some common gram‐positive and gram‐negative bacteria.

	5a	5b	5c	6a	6b	6c	7a	7b	7c	8a	8b	8c	Clor*	Str**
*S. aureus*	7500	3750	1875	3750	3750	3750	3750	3750	3750	15000	15000	7500	16	8
*E. faecalis*	1875	1875	1875	937.5	1875	1875	937.5	3750	1875	7500	7500	3750	256	256
*E. coli*	3750	3750	3750	3750	3750	3750	3750	3750	3750	7500	7500	7500	16	16
*K. pneumoniae*	3750	3750	3750	3750	3750	3750	3750	3750	3750	7500	7500	7500	128	256
*P. aeruginosa*	3750	3750	3750	3750	3750	3750	3750	3750	3750	7500	7500	7500	512	64

* Chloramphenicol.

** Streptomycin.

## 3 Experimental

### 3.1 Chemistry

#### 3.1.1 Synthesis of 2‐(((4‐(Prop‐1‐en‐2‐yl)cyclohex‐1‐en‐1‐yl)methoxy)methyl)oxirane (1)

**Scheme 2:**
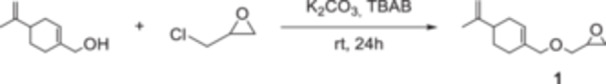
Synthesis scheme of 2‐(((4‐(prop‐1‐en‐2‐yl)cyclohex‐1‐en‐1‐yl)methoxy)methyl) oxirane (**1**).

In a 50 mL flask, (*S*)‐perillyl alcohol (1 mmol) was added to a mixture of epichlorohydrin (10 mmol), K_2_CO_3_ (2 mmol), and 10% TBAB. The reaction mixture was stirred at room temperature for 24 h. Then, epichlorohydrin was removed in a rotary evaporator. The residue was dissolved with 25 mL EtOAc and washed with water (2 × 25 mL). The organic phase was dried through magnesium sulfate and filtered, and the solvent was removed via vacuum. The crude product was purified by column chromatography with ethyl acetate: hexane (5:95) in 88% yield (Scheme 2) [38, 39, 40].

#### 3.1.2 General Synthesis of Oxirane Derivatives From Carvacrol (2), Eugenol (3), and Thymol (4)

**Scheme 3:**
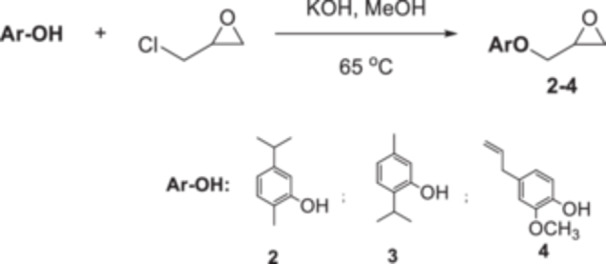
Synthesis of oxirane derivatives from carvacrol (**2**), thymol (**3**), and eugenol (**4**).

In a 50 mL flask, the phenolic compound (1 mmol) was dissolved in methanol (15 mL). KOH (2 mmol) and epichlorohydrin (10 mmol) were added. The mixture was stirred for 1 h at 65°C. After that, methanol was removed, and the residue was solved with ethyl acetate (25 mL) and washed with brine (25 mL). The collected organic phase was dried with magnesium sulfate and removed in a rotary evaporator. The resulting substances **2** and **3** were purified by column chromatography with hexane in 95% yield for **2** and 91% yield for **3,** while **4** was purified by crystallization from methanol with a 90% yield (Scheme 3) [32, 33, 34].

### 3.2 General Synthesis Procedure of 1,2‐Halohydrin Derivatives

**Scheme 4:**
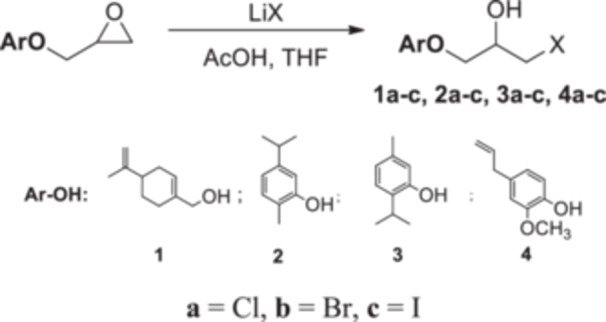
Synthesis procedure of 1,2‐halohydrin compounds.

In a 25 mL flask, acetic acid (3 mmol) and Li*X* (*X*: Cl, Br, I) (2 mmol) were added to the solution of oxirane compound (1 mmol) in tetrahydrofuran (10 mL), and the mixture was stirred at room temperature for 24 h. After that, water (25 mL) and ethyl acetate (25 mL) were added to the reaction mixture. The organic phase was separated, extracted with sodium thiosulfate (10%), and mixed with magnesium sulfate. The solvent was removed in a rotary evaporator, which yielded halohydrin products (Scheme 4).

#### 3.2.1 1‐Chloro‐3‐((4‐(prop‐1‐en‐2‐yl)cyclohex‐1‐en‐1‐yl)methoxy)propan‐2‐ol (1a)

Light yellow liquid, 85% yield; ^1^H NMR (300 MHz, CDCl_3_) *δ* 5.85–5.48 (m, 1H), 4.70 (dt, *J* = 4.0, 2.2 Hz, 2H), 4.04–3.80 (m, 3H), 3.72–3.52 (m, 2H), 3.46 (d, *J* = 5.1 Hz, 2H), 2.81 (s, 1H), 2.23–1.62 (m, 8H), 1.59–1.15 (m, 2H); ^13^C NMR (75 MHz, CDCl_3_) *δ* 149.85, 134.34, 125.42, 108.95, 75.98, 70.50, 70.31, 46.28, 41.21, 30.67, 27.58, 26.49, 20.99; HRMS (ESI) calculated for C_13_H_21_ClO_2_ *m/z* = 244.1230 found 245.1308 [M + H]^+^ (Schemes S1, S2, and S3).

#### 3.2.2 1‐Bromo‐3‐((4‐(prop‐1‐en‐2‐yl)cyclohex‐1‐en‐1‐yl)methoxy)propan‐2‐ol (1b)

Light orange liquid, 86% yield; ^1^H NMR (300 MHz, CDCl_3_) *δ* 5.89–5.58 (m, 1H), 4.94–4.58 (m, 2H), 4.03–3.75 (m, 3H), 3.64–3.33 (m, 4H), 2.83 (bs, 1H), 2.36–1.66 (m, 8H), 1.63–1.31 (m, 2H); ^13^C NMR (75 MHz, CDCl_3_) *δ* 149.85, 134.33, 125.46, 108.98, 76.00, 70.92, 70.16, 41.22, 35.38, 30.67, 27.59, 26.51, 21.02; HRMS (ESI) calculated for C_13_H_21_BrO_2_ *m/z* = 288.0725 found 289.0762 [M + H]^+^ (Schemes S4, S5, and S6).

#### 3.2.3 1‐Iodo‐3‐((4‐(prop‐1‐en‐2‐yl)cyclohex‐1‐en‐1‐yl)methoxy)propan‐2‐ol (1c)

Light orange liquid, 85% yield; ^1^H NMR (300 MHz, CDCl_3_) *δ* 5.67 (d, *J* = 6.3 Hz, 1H), 4.69 (d, *J* = 4.3 Hz, 2H), 3.86 (s, 2H), 3.73 (pd, *J* = 5.5, 1.9 Hz, 1H), 3.53–3.38 (m, 2H), 3.38–3.14 (m, 2H), 2.98 (bs, 1H), 2.28–1.60 (m, 8H), 1.57–1.07 (m, 2H); ^13^C NMR (75 MHz, CDCl_3_) *δ* 149.80, 134.32, 125.52, 109.03, 75.98, 72.11, 70.16, 41.21, 30.69, 27.61, 26.56, 21.07, 9.78; HRMS (ESI) calculated for C_13_H_21_IO_2_ *m/z* = 336.0586 found 337.0680 [M + H]^+^ (Schemes S7, S8, and S9).

#### 3.2.4 1‐Chloro‐3‐(5‐isopropyl‐2‐methylphenoxy)propan‐2‐ol (2a)

Light yellow liquid, 83% yield; ^1^H NMR (300 MHz, CDCl_3_) *δ* 7.09 (d, *J* = 7.7 Hz, 1H), 6.80 (d, *J* = 7.7 Hz, 1H), 6.73 (s, 1H), 4.29 (bs, 1H), 4.18–3.97 (m, 2H), 3.81 (qdd, *J* = 11.1, 5.5, 2.6 Hz, 2H), 3.01–2.78 (m, 1H), 2.70 (bs, 1H), 2.21 (s, 2H), 1.27 (d, *J* = 6.9 Hz, 6H); ^13^C NMR (75 MHz, CDCl_3_) *δ* 156.48, 148.44, 130.96, 124.31, 119.25, 110.11, 70.40, 68.83, 46.47, 34.39, 24.39 (2C), 16.07; HRMS (ESI) calculated for C_13_H_19_ClO_2_ *m/z* = 242.1074 found 242.1065 [M]^+^ (Schemes S10, S11, and S12).

#### 3.2.5 1‐Bromo‐3‐(5‐isopropyl‐2‐methylphenoxy)propan‐2‐ol (2b)

Light yellow liquid, 86% yield; ^1^H NMR (300 MHz, CDCl_3_) *δ* 7.11 (d, *J* = 7.6 Hz, 1H), 6.93–6.70 (m, 2H), 4.43–3.99 (m, 3H), 3.77–3.61 (m, 2H), 3.05–2.69 (m, 2H), 2.24 (s, 3H), 1.29 (d, *J* = 7.0 Hz, 6H); ^13^C NMR (75 MHz, CDCl_3_) *δ* 156.41, 148.44, 130.96, 124.29, 119.23, 110.06, 70.01, 69.40, 35.58, 34.39, 24.41 (2C), 16.12; HRMS (ESI) calculated for C_13_H_19_BrO_2_ *m/z* = 286.0568 found 286.0565 [M]^+^ (Schemes S13, S14, and S15).

#### 3.2.6 1‐Iodo‐3‐(5‐isopropyl‐2‐methylphenoxy)propan‐2‐ol (2c)

Light yellow liquid, 85% yield; ^1^H NMR (300 MHz, CDCl_3_) *δ* 7.18 (d, *J* = 7.7 Hz, 1H), 6.85 (d, *J* = 7.7 Hz, 1H), 6.74 (s, 1H), 4.22–3.95 (m, 3H), 3.66–3.42 (m, 2H), 3.42–3.18 (m, 1H), 2.89 (bs, 1H), 2.39 (s, 3H), 1.28 (d, *J* = 5.9 Hz, 6H); ^13^C NMR (75 MHz, CDCl_3_) *δ* 156.40, 148.42, 130.96, 124.31, 119.23, 110.13, 70.58, 70.07, 34.40, 24.46 (2C), 16.20, 9.79; HRMS (ESI) calculated for C_13_H_19_IO_2_ *m/z* = 334.0430 found 334.0426 [M]^+^ (Schemes S16, S17, and S18).

#### 3.2.7 1‐Chloro‐3‐(2‐isopropyl‐5‐methylphenoxy)propan‐2‐ol (3a)

Light yellow liquid, 80% yield; ^1^H NMR (300 MHz, CDCl_3_) *δ* 7.20 (d, *J* = 7.7 Hz, 1H), 6.88 (d, *J* = 7.8 Hz, 1H), 6.77 (s, 1H), 4.38–4.26 (m, 1H), 4.23–4.07 (m, 2H), 3.98–3.74 (m, 2H), 3.45–3.23 (m, 1H), 2.98 (bs, 1H), 2.52–2.28 (m, 3H), 1.33 (d, *J* = 7.0 Hz, 6H); ^13^C NMR (75 MHz, CDCl_3_) *δ* 155.46, 136.85, 134.32, 126.37, 122.33, 112.85, 70.42, 68.84, 46.59, 26.90, 23.16 (2C), 21.62; HRMS (ESI) calculated for C_13_H_19_ClO_2_ *m/z* = 242.1074 found 242.1066 [M]^+^ (Schemes S19, S20, and S21).

#### 3.2.8 1‐Bromo‐3‐(2‐isopropyl‐5‐methylphenoxy)propan‐2‐ol (3b)

Light yellow liquid, 84% yield; ^1^H NMR (300 MHz, CDCl_3_) *δ* 7.16 (d, *J* = 7.8 Hz, 1H), 6.84 (d, *J* = 7.8 Hz, 1H), 6.73 (s, 1H), 4.33–3.96 (m, 3H), 3.86–3.57 (m, 2H), 3.30 (hept, *J* = 6.9 Hz, 1H), 2.79 (bs, 1H), 2.37 (s, 3H), 1.26 (d, *J* = 6.9 Hz, 6H); ^13^C NMR (75 MHz, CDCl_3_) *δ* 155.35, 136.84, 134.25, 126.33, 122.28, 112.77, 69.97, 69.41, 35.68, 26.86, 23.13, 21.61; HRMS (ESI) calculated for C_13_H_19_BrO_2_ *m/z* = 286.0568 found 286.0562 [M]^+^ (Schemes S22, S23, and S24).

#### 3.2.9 1‐Iodo‐3‐(2‐isopropyl‐5‐methylphenoxy)propan‐2‐ol (3c)

Light yellow liquid, 84% yield; ^1^H NMR (300 MHz, CDCl_3_) *δ* 7.17 (d, *J* = 7.8 Hz, 1H), 6.85 (d, *J* = 7.8 Hz, 1H), 6.74 (s, 1H), 4.21–3.94 (m, 3H), 3.59–3.41 (m, 2H), 3.39–3.20 (m, 1H), 2.89 (bs, 1H), 2.38 (s, 3H), 1.27 (d, *J* = 7.0 Hz, 6H); ^13^C NMR (75 MHz, CDCl_3_) δ 155.35, 136.84, 134.26, 126.35, 122.31, 112.83, 70.66, 70.00, 26.89, 23.21(2C), 21.68, 10.04; HRMS (ESI) calculated for C_13_H_19_IO_2_ *m/z* = 334.0430 found 334.0424 [M]^+^ (Schemes S25, S26, and S27).

#### 3.2.10 1‐(4‐Allyl‐2‐methoxyphenoxy)‐3‐chloropropan‐2‐ol (4a)

Light yellow liquid, 79% yield; ^1^H NMR (300 MHz, CDCl_3_) *δ* 6.98–6.82 (m, 1H), 6.79–6.68 (m, 2H), 6.10–5.85 (m, 1H), 5.17–4.99 (m, 2H), 4.18 (q, *J* = 5.3 Hz, 1H), 4.09 (t, *J* = 4.4 Hz, 2H), 3.93–3.64 (m, 6H), 3.34 (d, *J* = 6.7 Hz, 2H); ^13^C NMR (75 MHz, CDCl_3_) *δ* 149.85, 146.43, 137.73, 134.57, 121.08, 116.06, 115.49, 112.75, 71.43, 70.16, 56.08, 45.77, 40.07; HRMS (ESI) calculated for C_13_H_17_ClO_3_ *m/z* = 256.0866 found 279.0755 [M+Na]^+^ (Schemes 28, S29, and S30).

#### 3.2.11 1‐(4‐Allyl‐2‐methoxyphenoxy)‐3‐bromopropan‐2‐ol (4b)

Light yellow liquid, 80% yield; ^1^H NMR (300 MHz, CDCl_3_) *δ* 6.87 (d, *J* = 8.6 Hz, 1H), 6.79–6.64 (m, 2H), 6.12–5.82 (m, 1H), 5.22–4.92 (m, 2H), 4.28–4.03 (m, 3H), 3.84 (s, 3H), 3.71–3.48 (m, 3H), 3.43–3.19 (m, 2H); ^13^C NMR (75 MHz, CDCl_3_) *δ* 149.88, 146.35, 137.71, 134.63, 121.04, 116.11, 115.63, 112.67, 72.14, 69.78, 56.08, 40.10, 34.67; HRMS (ESI) calculated for C_13_H_17_BrO_3_ *m/z* = 300.0361 found 323.0253 [M+Na]^+^ (Schemes S31, S32, and S33).

#### 3.2.12 1‐(4‐Allyl‐2‐methoxyphenoxy)‐3‐iodopropan‐2‐ol (4c)

Light yellow liquid, 79% yield; ^1^H NMR (300 MHz, CDCl_3_) *δ* 6.87 (d, *J* = 8.6 Hz, 1H), 6.81–6.62 (m, 2H), 5.95 (ddt, *J* = 15.5, 10.4, 6.7 Hz, 1H), 5.21–4.96 (m, 2H), 4.07 (dd, *J* = 4.9, 1.6 Hz, 2H), 4.04–3.91 (m, 1H), 3.84 (d, *J* = 2.9 Hz, 3H), 3.63–3.20 (m, 5H); ^13^C NMR (75 MHz, CDCl_3_) *δ* 150.01, 146.36, 137.69, 134.79, 121.06, 116.12, 116.01, 112.72, 73.55, 69.78, 56.12, 40.11, 8.65; HRMS (ESI) calculated for C_13_H_17_IO_3_ *m/z* = 348.0222 found 371.0112 [M+Na]^+^ (Schemes S34, S35, and S36).

### 3.3 General Synthesis Procedure of Thiran Derivatives (5, 6, 7, 8)

**Scheme 5:**
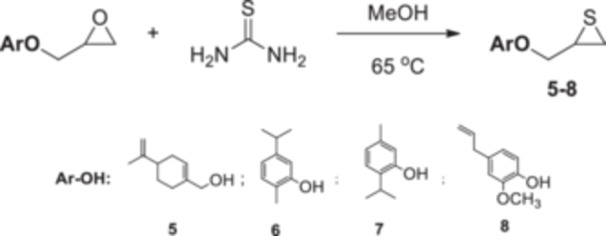
The synthesis procedure of thiran derivatives (**5**, **6**, **7**, **8**).

Thiourea (2 mmol) was added to the solution of the oxirane (1 mmol) in methanol (15 mL). After stirring at 65°C for 1.5 h, methanol was removed from a rotary evaporator. The crude product was dissolved with dichloromethane (25 mL) and extracted with brine (25 mL). The organic phase was dried via magnesium sulfate and removed by a rotary evaporator. The obtained product was used in further reaction without further purification (**5** 92%, **6** 94%, **7** 94%, **8** 93%) (Scheme 5).

### 3.4 General Synthesis Procedure of β‐Halo Thiol Compounds

**Scheme 6:**
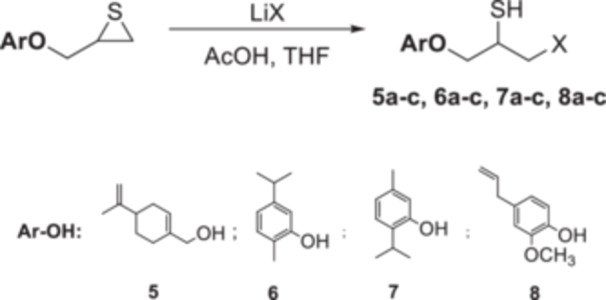
Synthesis procedure of β‐halo thiol compounds.

In a 25 mL flask, acetic acid (3 mmol) and Li*X* (*X*: Cl, Br, I) (2 mmol) were added to this solution of thiran compound (1 mmol) in tetrahydrofuran (10 mL), and the mixture was stirred at room temperature for 24 h. After that, water (25 mL) and ethyl acetate (25 mL) were added to the reaction mixture. The organic phase was separated, extracted with sodium thiosulfate (10%), and mixed with magnesium sulfate. The solvent was removed in a rotary evaporator, which yielded halohydrin products (Scheme 6).

#### 3.4.1 1‐Chloro‐3‐((4‐(prop‐1‐en‐2‐yl)cyclohex‐1‐en‐1‐yl)methoxy)propane‐2‐thiol (5a)

Light orange liquid, 83% yield; ^1^H NMR (300 MHz, CDCl_3_) *δ* 5.73–5.67 (m, 1H), 4.70 (d, *J* = 4.0 Hz, 2H), 3.90 (s, 2H), 3.58 (dd, *J* = 10.7, 5.6 Hz, 1H), 3.36 (dd, *J* = 10.6, 6.8 Hz, 1H), 3.06 (p, *J* = 6.0 Hz, 1H), 2.51 (d, *J* = 6.2 Hz, 1H), 2.27–1.79 (m, 7H), 1.72 (s, 3H), 1.47 (m, 2H); ^13^C NMR (75 MHz, CDCl_3_) *δ* 149.94, 134.60, 125.26, 108.93, 75.67, 74.40, 41.24, 32.52, 30.71, 27.63, 26.58, 24.19, 21.03; HRMS (ESI) calculated for C_13_H_21_ClOS *m/z* = 260.1002 found 261.1073 [M + H]^+^ (Schemes S37, S38, and S39).

#### 3.4.2 1‐Bromo‐3‐((4‐(prop‐1‐en‐2‐yl)cyclohex‐1‐en‐1‐yl)methoxy)propane‐2‐thiol (5b)

Light orange liquid, 83% yield; ^1^H NMR (300 MHz, CDCl_3_) *δ* 5.84–5.56 (m, 1H), 4.70 (d, *J* = 4.2 Hz, 2H), 3.90 (s, 2H), 3.58 (dd, *J* = 10.7, 5.7 Hz, 1H), 3.36 (dd, *J* = 10.6, 6.9 Hz, 1H), 3.06 (p, *J* = 5.9 Hz, 1H), 2.51 (d, *J* = 6.2 Hz, 1H), 2.33–1.77 (m, 7H), 1.72 (s, 3H), 1.57–1.37 (m, 2H); ^13^C NMR (75 MHz, CDCl_3_) *δ* 149.94, 134.61, 125.25, 108.93, 75.69, 74.41, 41.27, 32.53, 30.71, 27.63, 26.58, 24.19, 21.03; HRMS (ESI) calculated for C_13_H_21_BrOS‐HBr *m/z* = 225.1308 found 225.1314 [M‐HBr]^+^ (Schemes S40, S41, and S42).

#### 3.4.3 1‐Iodo‐3‐((4‐(prop‐1‐en‐2‐yl)cyclohex‐1‐en‐1‐yl)methoxy)propane‐2‐thiol (5c)

Light orange liquid, 84% yield; ^1^H NMR (300 MHz, CDCl_3_) *δ* 5.76–5.57 (m, 1H), 4.69 (d, *J* = 3.9 Hz, 2H), 3.89 (s, 2H), 3.57 (dd, *J* = 10.6, 5.6 Hz, 1H), 3.35 (dd, *J* = 10.6, 6.8 Hz, 1H), 3.06 (h, *J* = 6.8, 6.2 Hz, 1H), 2.50 (d, *J* = 6.2 Hz, 1H), 2.30–1.78 (m, 7H), 1.71 (s, 3H), 1.59–1.36 (m, 2H); ^13^C NMR (75 MHz, CDCl_3_) *δ* 149.89, 134.59, 125.23, 108.94, 75.67, 74.39, 41.26, 32.51, 30.70, 27.63, 26.57, 24.17, 21.03; HRMS (ESI) calculated for C_13_H_21_IOS‐HI *m/z* = 225.1308 found 225.1315 [M‐HI]^+^ (Schemes S43, S44, and S45).

#### 3.4.4 1‐Chloro‐3‐(5‐isopropyl‐2‐methylphenoxy)propane‐2‐thiol (6a)

Light yellow liquid, 81% yield; ^1^H NMR (300 MHz, CDCl_3_) *δ* 7.21 (d, *J* = 7.6 Hz, 1H), 6.91 (d, *J* = 7.6 Hz, 1H), 6.84 (s, 1H), 4.37 (dd, *J* = 10.3, 5.4 Hz, 1H), 4.18–3.97 (m, 1H), 3.50–3.29 (m, 1H), 3.16–2.88 (m, 1H), 2.72 (d, *J* = 6.2 Hz, 1H), 2.51–2.27 (m, 5H), 1.40 (d, *J* = 6.9 Hz, 6H); ^13^C NMR (75 MHz, CDCl_3_) *δ* 156.90, 148.31, 131.04, 124.70, 119.15, 110.44, 73.13, 34.52, 32.13, 24.57 (2C), 24.35, 16.30; HRMS (ESI) calculated for C_13_H_19_ClOS‐HCl *m/z* = 223.1151 found 223.1155 [M‐HCl]^+^ (Schemes S46, S47, and S48).

#### 3.4.5 1‐Bromo‐3‐(5‐isopropyl‐2‐methylphenoxy)propane‐2‐thiol (6b)

Light yellow liquid, 82% yield; ^1^H NMR (300 MHz, CDCl_3_) *δ* 7.14 (d, *J* = 7.6 Hz, 1H), 6.84 (d, *J* = 7.6 Hz, 1H), 6.75 (s, 1H), 4.31 (dd, *J* = 10.2, 5.4 Hz, 1H), 3.99 (dd, *J* = 10.2, 7.0 Hz, 1H), 3.44–3.27 (m, 1H), 2.93 (hept, *J* = 6.9 Hz, 1H), 2.67 (t, *J* = 6.2 Hz, 1H), 2.43–2.36 (m, 1H), 2.35–2.23 (m, 4H), 1.31 (d, *J* = 6.9 Hz, 6H); ^13^C NMR (75 MHz, CDCl_3_) δ 156.80, 148.27, 130.95, 124.65, 119.07, 110.36, 73.05, 34.43, 32.05, 24.48 (2C), 24.37, 24.33, 16.20; HRMS (ESI) calculated for C_13_H_19_BrOS‐HBr *m/z* = 223.1151 found 223.1155 [M‐HBr]^+^ (Schemes S49, S50, and S51).

#### 3.4.6 1‐Iodo‐3‐(5‐isopropyl‐2‐methylphenoxy)propane‐2‐thiol (6c)

Light yellow liquid, 82% yield; ^1^H NMR (300 MHz, CDCl_3_) *δ* 7.15 (d, *J* = 7.6 Hz, 1H), 6.85 (dd, *J* = 7.6, 1.8 Hz, 1H), 6.76 (d, *J* = 1.8 Hz, 1H), 4.31 (dd, *J* = 10.3, 5.4 Hz, 1H), 4.00 (dd, *J* = 10.3, 7.0 Hz, 1H), 3.50–3.27 (m, 1H), 2.94 (p, *J* = 7.0 Hz, 1H), 2.67 (dd, *J* = 6.2, 1.2 Hz, 1H), 2.44–2.22 (m, 5H), 1.32 (d, *J* = 7.0 Hz, 6H); ^13^C NMR (75 MHz, CDCl_3_) *δ* 156.82, 148.27, 130.96, 124.66, 119.09, 110.39, 73.08, 34.44, 32.06, 24.49 (2C), 24.32, 16.21; HRMS (ESI) calculated for C_13_H_19_IOS‐HI *m/z* = 223.1151 found 223.1152 [M‐HI]^+^ (Schemes S52, S53, and S54).

#### 3.4.7 1‐Chloro‐3‐(2‐isopropyl‐5‐methylphenoxy)propane‐2‐thiol (7a)

Light yellow liquid, 80% yield; ^1^H NMR (300 MHz, CDCl_3_) *δ* 7.15 (d, *J* = 7.6 Hz, 1H), 6.85 (dt, *J* = 7.6, 1.9 Hz, 1H), 6.76 (d, *J* = 1.9 Hz, 1H), 4.31 (dd, *J* = 10.3, 5.3 Hz, 1H), 4.06–3.93 (m, 1H), 3.47–3.22 (m, 1H), 3.02–2.83 (m, 1H), 2.67 (d, *J* = 6.2 Hz, 1H), 2.47–2.23 (m, 5H), 1.32 (d, *J* = 7.0 Hz, 6H); ^13^C NMR (75 MHz, CDCl_3_) *δ* 156.83, 148.27, 130.96, 124.67, 119.10, 110.41, 73.09, 34.43, 32.05, 24.48 (2 C), 24.30, 16.19; HRMS (ESI) calculated for C_13_H_19_ClOS‐HCl *m/z* = 223.1151 found 223.1154 [M‐HCl]^+^ (Schemes S55, S56, and S57).

#### 3.4.8 1‐Bromo‐3‐(2‐isopropyl‐5‐methylphenoxy)propane‐2‐thiol (7b)

Light yellow liquid, 81% yield; ^1^H NMR (300 MHz, CDCl_3_) *δ* 7.17 (d, *J* = 7.7 Hz, 1H), 6.83 (d, *J* = 7.7 Hz, 1H), 6.70 (s, 1H), 4.25 (dd, *J* = 10.2, 5.6 Hz, 1H), 3.99 (dd, *J* = 10.2, 6.8 Hz, 1H), 3.51–3.26 (m, 2H), 2.66 (d, *J* = 6.2 Hz, 1H), 2.51–2.27 (m, 5H), 1.29 (d, *J* = 7.0 Hz, 6H); ^13^C NMR (75 MHz, CDCl_3_) *δ* 155.80, 136.66, 134.57, 126.41, 122.06, 113.03, 73.01, 32.03, 27.01, 24.13, 23.15 (2C), 23.11, 21.62; HRMS (ESI) calculated for C_13_H_19_BrOS‐HBr *m/z* = 223.1151 found 223.1153 [M‐HBr]^+^ (Schemes S58, S59, and S60).

#### 3.4.9 1‐Iodo‐3‐(2‐isopropyl‐5‐methylphenoxy)propane‐2‐thiol (7c)

Light yellow liquid, 82% yield; ^1^H NMR (300 MHz, CDCl_3_) *δ* 7.16 (d, *J* = 7.8 Hz, 1H), 6.82 (dd, *J* = 7.8, 1.7 Hz, 1H), 6.69 (d, *J* = 1.7 Hz, 1H), 4.24 (dd, *J* = 10.2, 5.5 Hz, 1H), 3.98 (dd, *J* = 10.3, 6.9 Hz, 1H), 3.44–3.25 (m, 2H), 2.65 (d, *J* = 6.2 Hz, 1H), 2.43–2.17 (m, 4H), 1.28 (d, *J* = 7.0 Hz, 7H); ^13^C NMR (75 MHz, CDCl_3_) *δ* 155.80, 136.65, 134.57, 126.40, 122.05, 113.02, 73.00, 32.02, 26.99, 24.13, 23.14, 23.09, 21.60; HRMS (ESI) calculated for C_13_H_19_IOS‐HI *m/z* = 223.1151 found 223.1154 [M‐HI]^+^ (Schemes S61, S62, and S63).

#### 3.4.10 1‐(4‐Allyl‐2‐methoxyphenoxy)‐3‐chloropropane‐2‐thiol (8a)

Light yellow liquid, 86% yield; ^1^H NMR (300 MHz, CDCl_3_) *δ* 6.84 (d, *J* = 7.9 Hz, 1H), 6.79–6.64 (m, 2H), 6.12–5.83 (m, 1H), 5.16–4.97 (m, 2H), 4.30 (dd, *J* = 10.5, 5.1 Hz, 1H), 3.96–3.69 (m, 5H), 3.41–3.18 (m, 3H), 2.59 (d, *J* = 6.2 Hz, 1H), 2.32 (d, *J* = 5.3 Hz, 1H); ^13^C NMR (75 MHz, CDCl_3_) *δ* 149.86, 146.35, 137.78, 134.23, 120.76, 116.02, 114.99, 112.67, 74.54, 56.10, 40.09, 31.67, 24.77; HRMS (ESI) calculated for C_13_H_17_ClO_2_S‐HCl *m/z* = 237.0944 found 237.0947 [M‐HCl]^+^ (Schemes S64, S65, and S66).

#### 3.4.11 1‐(4‐Allyl‐2‐methoxyphenoxy)‐3‐bromopropane‐2‐thiol (8b)

Light yellow liquid, 87% yield; ^1^H NMR (300 MHz, CDCl_3_) *δ* 6.84 (d, *J* = 8.0 Hz, 1H), 6.77– 6.65 (m, 2H), 6.09–5.86 (m, 1H), 5.21–4.97 (m, 2H), 4.30 (dd, *J* = 10.6, 5.1 Hz, 1H), 3.97–3.70 (m, 5H), 3.41–3.23 (m, 3H), 2.59 (d, *J* = 6.1 Hz, 1H), 2.31 (d, *J* = 5.3 Hz, 1H); ^13^C NMR (75 MHz, CDCl_3_) *δ* 149.85, 146.35, 137.79, 134.21, 120.76, 116.03, 114.97, 112.66, 74.53, 56.09, 40.09, 31.68, 24.76; HRMS (ESI) calculated for C_13_H_17_BrO_2_S‐HBr *m/z* = 237.0944 found 237.0948 [M‐HBr]^+^ (Schemes S67, S68, and S68).

#### 3.4.12 1‐(4‐Allyl‐2‐methoxyphenoxy)‐3‐iodopropane‐2‐thiol (8c)

Light yellow liquid, 87% yield; ^1^H NMR (300 MHz, CDCl_3_) *δ* 6.84 (d, *J* = 8.0 Hz, 1H), 6.79–6.61 (m, 2H), 6.11–5.82 (m, 1H), 5.15–5.00 (m, 2H), 4.30 (dd, *J* = 10.6, 5.1 Hz, 1H), 4.00–3.74 (m, 5H), 3.42–3.22 (m, 3H), 2.59 (d, *J* = 6.2 Hz, 1H), 2.32 (d, *J* = 5.2 Hz, 1H); ^13^C NMR (75 MHz, CDCl_3_) *δ* 149.86, 146.35, 137.78, 134.23, 120.75, 116.04, 114.96, 112.66, 74.54, 56.11, 40.10, 31.68, 24.80; HRMS (ESI) calculated for C_13_H_17_IO_2_S‐HI *m/z* = 237.0944 found 237.0947 [M‐HI]^+^ (Schemes S70, S71, and S72).

### 3.5 Antibacterial Studies

In this in vitro study, five standard bacterial strains (*E. coli*‐ATCC 25922, *P. aeruginosa*‐ATCC 27853, *K. pneumoniae*‐ATTC 700603, *S. aureus*‐ATCC 29213, *Enterococcus feacalis*‐ATTC 29212) were used to evaluate the antimicrobial activity of the synthesized chemical compounds using the microdilution method.

To revive the test microorganisms, they were passaged into Mueller Hinton broth (MHB) medium on the first day and into Mueller Hinton agar (MHA) the next day and incubated at 37°C for 18 h. Colonies grown on MHA were collected using a sterile cotton swab and inoculated into sterile physiological saline, and the suspension was prepared to a 0.5 McFarland standard. The prepared bacterial suspension was diluted 1/20 with sterile physiological saline and inoculated into microplate wells within 15 min.

U‐bottom, 96‐well, sterile microplates (Isolab, Akron, Ohio) were used for the broth microdilution study. Fifty microliters of the freshly prepared MHB medium was distributed to all wells. Fifty microliters of the chemicals to be tested were added to the wells in the first row, and serial dilution continued by taking 50 µL and transferring it to the next well between the first well and the seventh well. The last 50 µL of liquid drawn into the pipette was thrown out. No chemicals were added to the eighth well because it would be used as a growth control. Five microliters of the prepared microorganism suspension was distributed to all wells, ensuring that the final microorganism inoculum in the wells was ~5 × 10^5^ CFU/mL. In addition, mixtures of each chemical with MHB were mixed and tested in another well to ensure sterility. The microdilution plates were incubated in ambient air at 37°C for 24 h. After incubation, the results were evaluated, and the lowest concentration at which growth was inhibited was determined as the MIC and recorded. The average MIC values were calculated in duplicate wells, and the well containing the higher concentration of chemicals was accepted as the MIC [41].

All β‐halo alcohols have equal inhibitor activity against *S. aureus* except the derivative prepared from carvacrol and chlorine (**2a**), which has a relatively lower activity. Derivatives prepared from perillyl alcohol (**1a–c**) showed equal activity against *S. aureus*. While the iodinated derivative **1c** showed the lowest activity against *E. faecalis* and *P. aeruginosa*, the brominated derivative **1b** showed the lowest activity against *E. coli*. On the other hand, the iodinated derivative **1c** also showed the highest inhibitory activity against *K. pneumoniae*.

It can be said that the inhibitory properties of the compounds prepared from the structural isomers carvacrol (**2a–c**) and thymol (**3a–c**) are similar. They have the same activity against *S. aureus* except for the derivative prepared from carvacrol and chlorine (**2a**).

All derivatives prepared from carvacrol (**2a–c**) and thymol (**3a–c**) have equal activity against *E. faecalis*. While chlorinated derivatives (**2a** and **3a**) and the compound prepared from thymol and iodine (**3c**) were more active against *E. coli*, the derivative prepared from thymol and bromine (**3b**) was relatively the most active against *K. pneumoniae*. For *P. aeruginosa*, the best‐performing compounds were those prepared from carvacrol and chlorine (**2a**) and compounds prepared with carvacrol or thymol and bromine (**2b**, **3b**), and the least‐performing compounds were the ones prepared with thymol and chlorine (**3a**).

In general, it can be said that eugenol derivatives (**4a–c**) work the worst against all bacteria. They (**4a–c**) showed equal activity against *S. aureus*. Brominated derivative (**4b**) worked the best against *E. faecalis* and *E. coli* among **4a–c**. Chlorinated derivative (**4a**) worked the worst against *K. pneumoniae* and *P. aeruginosa*, while brominated (**4b**) and iodinated (**4c**) derivatives gave equal results.

As mentioned before, lower inhibitory effects were observed in β‐halo thiols. Among the derivatives prepared with perillyl alcohol, the one that worked best against *S. aureus* was a **5c** compound prepared with iodine. No difference was observed between the activities against other bacteria.

It is seen that similar results were obtained in carvacrol and thymol isomers. While the activities against *S. aureus*, *E. coli*, *K. pneumoniae*, and *P. aeruginosa* were the same, the only difference was observed against *E. faecalis*. In that case, chlorinated derivatives **6a** and **7a** worked relatively best, while the worst was obtained with **7b** prepared with thymol and bromine. As in β‐halo alcohols, the inhibitory effects of eugenol derivatives are the worst among them. The derivatives prepared with chlorine and bromine showed lower inhibitory effects against *S. aureus* than all prepared derivatives. Although the highest effect was obtained with **8c** prepared with iodine against *E. faecalis*, it was not significant compared to Chloramphenicol and Streptomycin.

## 4 Conclusion

In this study, 12 new β‐halo alcohols and 12 new β‐halo thiol derivatives were designed, synthesized, and characterized. Natural alcohol derivatives, whose pharmacological effects have been known for a long time, were chosen as starting materials. In this way, it was aimed to synthesize product structures with high potential as pharmaceutical active ingredients. β‐halo alcohol derivatives were synthesized in two steps, and β‐halo thiol derivatives were synthesized in three steps. The β‐halo alcohol derivatives were synthesized with yields ranging from 79% to 82%, while the β‐halo thiol derivatives were obtained with yields between 66% and 71%, resulting in very high total yields.

The 24 new derivatives were compared with the commercially available Chloramphenicol and Streptomycin antibiotics. As a result, it was determined that the antibacterial activities of the new β‐halo alcohol and β‐halo thiols were not at the expected level. On the other hand, it has been observed that β‐halo alcohols generally have better antibacterial effects than β‐halo thiols. This effect can be attributed to the presence of oxygen in place of sulfur. When the results obtained with derivatives containing only sulfur instead of oxygen were compared, no correlation was observed.

Interest in organohalogen compounds began with the discovery of valuable ocean chemicals. Developing novel derivatives of halogenated phenoxypropanols, known to be involved in biological processes, is a relatively new field for organic chemistry. On the other hand, studies on phenoxypropane thiols are quite limited. For this reason, it has been contributed to the literature by developing halogenated phenoxypropanols (β‐halo alcohols) and phenoxypropane thiol (β‐halo thiol) derivatives and examining their biological activities.

## Author Contributions

**Fatma Çakmak:** investigation, writing – original draft. **Hande Toptan:** investigation, writing – original draft, methodology, visualization, writing – review and editing, conceptualization, validation, supervision, project administration. **Hayriye Genc Bilgicli:** conceptualization, investigation, writing – original draft, writing – review and editing, methodology, validation, visualization, supervision, project administration. **Mehmet Köroğlu:** supervision, data curation, visualization, validation, methodology, conceptualization, investigation, writing – original draft, project administration. **Mustafa Zengin:** conceptualization, investigation, writing – original draft, methodology, validation, data curation, supervision.

## Supporting information

Supporting information.

## Data Availability

The data that support the findings of this study are available in the Supporting material of this article.
