# Supplementary material for: Synthesis and Investigation of Antibacterial Properties of Thymol, Carvacrol, Eugenol, and Perillyl Alcohol Based β‐Halo Alcohol and β‐Halo Thiol Compounds
Source: J Biochem Mol Toxicol. 2025 Feb 17;39(2):e70171. doi: 10.1002/jbt.70171 (PMC11831588; doi:10.1002/jbt.70171)
Supplement: Supplementary file 1 — Supporting information. [file JBT-39-e70171-s001.docx]

**Synthesis and Investigation of Antibacterial Properties of Thymol, Carvacrol, Eugenol and Perillyl Alcohol Based β-halo Alcohol and β-halo Thiol Compounds**

**Fatma Diler^1^, Hande Toptan^2^, Hayriye Genç Bilgiçli*^,1^, Mehmet Köroğlu^3^, Mustafa Zengin^1^**

*^1^Sakarya University, Science Faculty, Chemistry Department, 54187, Sakarya/TÜRKİYE*

*^2^Sakarya University Training and Research Hospital, Medical Microbiology, Sakarya, TÜRKİYE*

*^3^Sakarya University, Medicine Faculty, Clinical Microbiology Department, Sakarya, TÜRKİYE*

Scheme 1. ^1^H NMR spectrum of compound **1a** (300 MHz, CDCl₃)

Scheme 2. ^13^C NMR spectrum of compound **1a** (75 MHz, CDCl₃)

Scheme 3. HRMS (ESI) calculated for **1a** C_13_H_21_ClO_2_ m/z = 244.1230 found 245.1308 [M+H]^+^

Scheme 4. ^1^H NMR spectrum of compound **1b** (300 MHz, CDCl₃)

Scheme 5. ^13^C NMR spectrum of compound **1b** (75 MHz, CDCl₃)

Scheme 6. HRMS (ESI) calculated for C_13_H_21_BrO_2_ m/z = 288.0725 found 289.0762 [M+H]^+^.


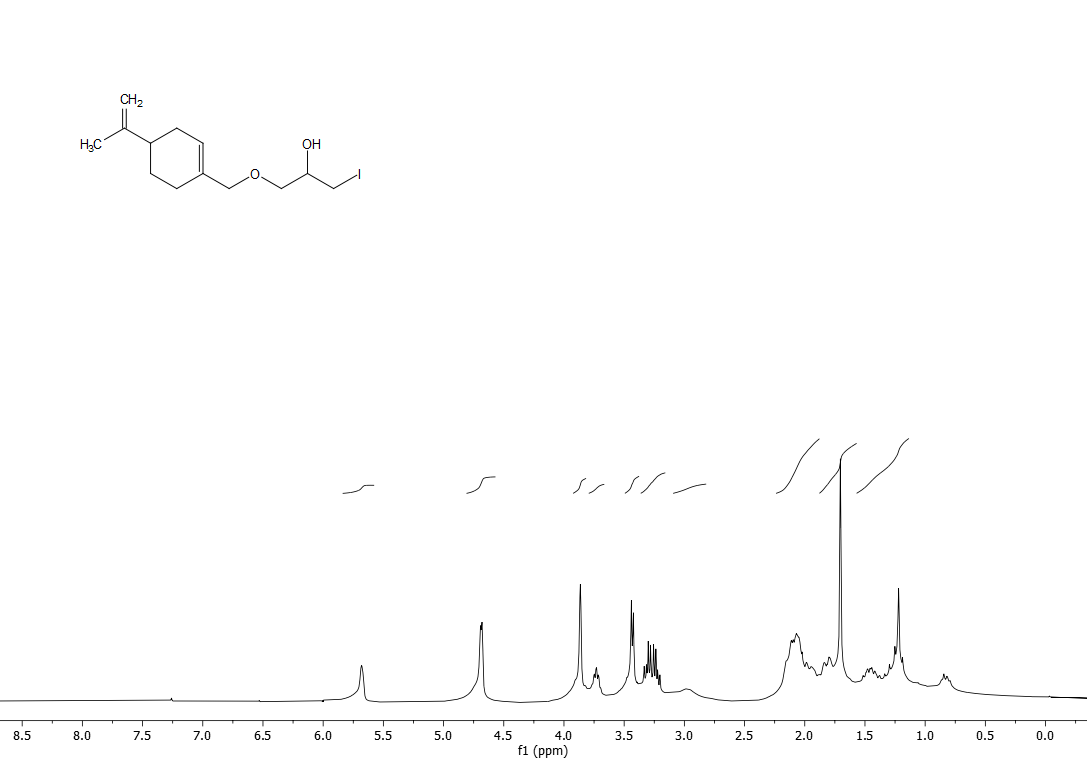


Scheme 7. ^1^H NMR spectrum of compound **1c** (300 MHz, CDCl₃)

Scheme 8. ^13^C NMR spectrum of compound **1c** (75 MHz, CDCl₃)

Scheme 9. HRMS (ESI) calculated for C_13_H_21_IO_2_ m/z = 336.0586 found 337.0680 [M+H]^+^.

Scheme 10. ^1^H NMR spectrum of compound **2a** (300 MHz, CDCl₃)

Scheme 11. ^13^C NMR spectrum of compound **2a** (75 MHz, CDCl₃)

Scheme 12. HRMS (ESI) calculated for C_13_H_19_ClO_2_ *m/z* = 242.1074 found 242.1065 [M]^+^.

Scheme 13. ^1^H NMR spectrum of compound **2b** (300 MHz, CDCl₃)

Scheme 14. ^13^C NMR spectrum of compound **2b** (75 MHz, CDCl₃)

Scheme 15. HRMS (ESI) calculated for C_13_H_19_BrO_2_ *m/z* = 286.0568 found 286.0565 [M]^+^.

Scheme 16. ^1^H NMR spectrum of compound **2c** (300 MHz, CDCl₃)

Scheme 17. ^13^C NMR spectrum of compound **2c** (75 MHz, CDCl₃)

Scheme 18. HRMS (ESI) calculated for C_13_H_19_IO_2_ *m/z* = 334.0430 found 334.0426 [M]^+^.

Scheme 19. ^1^H NMR spectrum of compound **3a** (300 MHz, CDCl₃)

Scheme 20. ^13^C NMR spectrum of compound **3a** (75 MHz, CDCl₃)

Scheme 21. HRMS (ESI) calculated for C_13_H_19_ClO_2_ *m/z* = 242.1074 found 242.1066 [M]^+^

Scheme 22. ^1^H NMR spectrum of compound **3b** (300 MHz, CDCl₃)

Scheme 23. ^13^C NMR spectrum of compound **3b** (75 MHz, CDCl₃)

Scheme 24. HRMS (ESI) calculated for C_13_H_19_BrO_2_ *m/z* = 286.0568 found 286.0562 [M]^+^.

Scheme 25. ^1^H NMR spectrum of compound **3c** (300 MHz, CDCl₃)

Scheme 26. ^13^C NMR spectrum of compound **3c** (75 MHz, CDCl₃)

Scheme 27. HRMS (ESI) calculated for C_13_H_19_IO_2_ *m/z* = 334.0430 found 334.0424 [M]^+^

Scheme 28. ^1^H NMR spectrum of compound **4a** (300 MHz, CDCl₃)

Scheme 29. ^13^C NMR spectrum of compound **4a** (75 MHz, CDCl₃)

Scheme 30. HRMS (ESI) calculated for C_13_H_17_ClO_3_ *m/z* = 256.0866 found 279.0755 [M+Na]^+^.

Scheme 31. ^1^H NMR spectrum of compound **4b** (300 MHz, CDCl₃)

Scheme 32. ^13^C NMR spectrum of compound **4b** (75 MHz, CDCl₃)

Scheme 33. HRMS (ESI) calculated for C_13_H_17_BrO_3_ *m/z* = 300.0361 found 323.0253 [M+Na]^+^.

Scheme 34. ^1^H NMR spectrum of compound **4c** (300 MHz, CDCl₃)

Scheme 35. ^13^C NMR spectrum of compound **4c** (75 MHz, CDCl₃)

Scheme 36. HRMS (ESI) calculated for C_13_H_17_IO_3_ *m/z* = 348.0222 found 371.0112 [M+Na]^+^

Scheme 37. ^1^H NMR spectrum of compound **5a** (300 MHz, CDCl₃)

Scheme 38. ^13^C NMR spectrum of compound **5a** (75 MHz, CDCl₃)

Scheme 39. HRMS (ESI) calculated for C_13_H_21_ClOS *m/z* = 260.1002 found 261.1073 [M+H]^+^

Scheme 40. ^1^H NMR spectrum of compound **5b** (300 MHz, CDCl₃)

Scheme 41. ^13^C NMR spectrum of compound **5b** (75 MHz, CDCl₃)

**
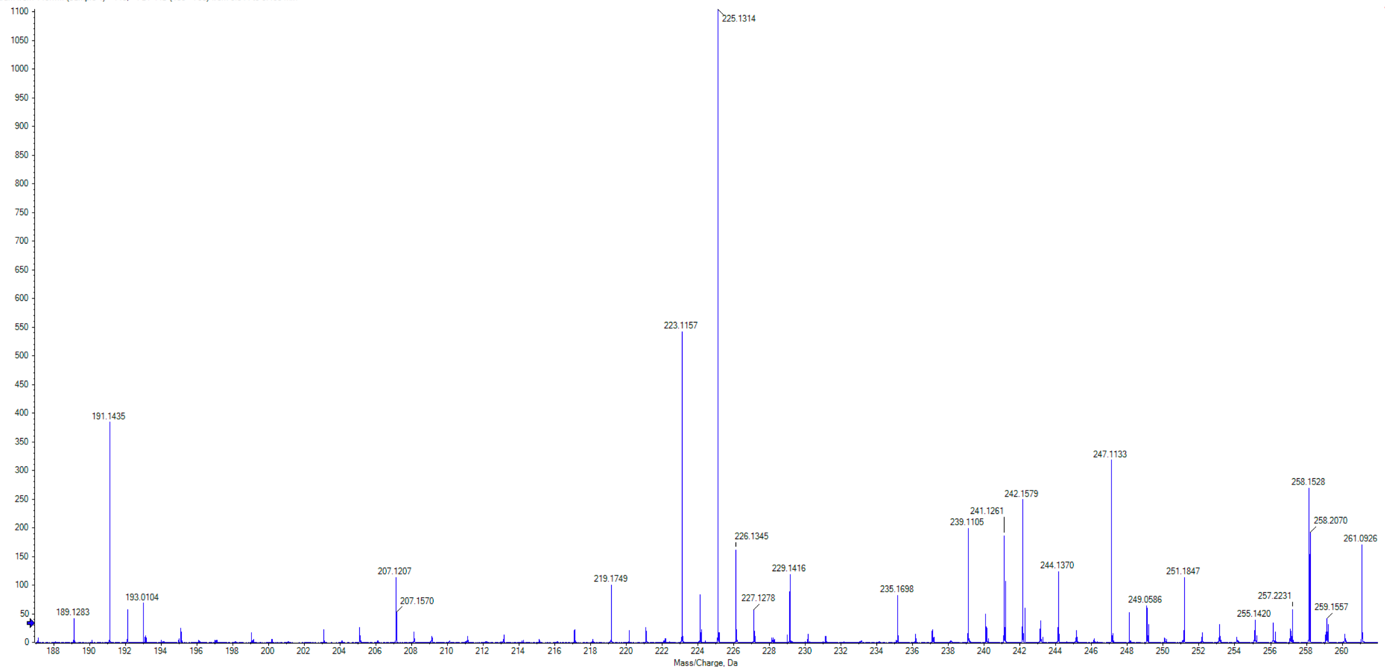
**

Scheme 42. HRMS (ESI) calculated for C_13_H_21_BrOS-HBr *m/z* = 224.1235 found 225.1314 [M-HBr]^+^

Scheme 43. ^1^H NMR spectrum of compound **5c** (300 MHz, CDCl₃)

Scheme 44. ^13^C NMR spectrum of compound **5c** (75 MHz, CDCl₃)

**
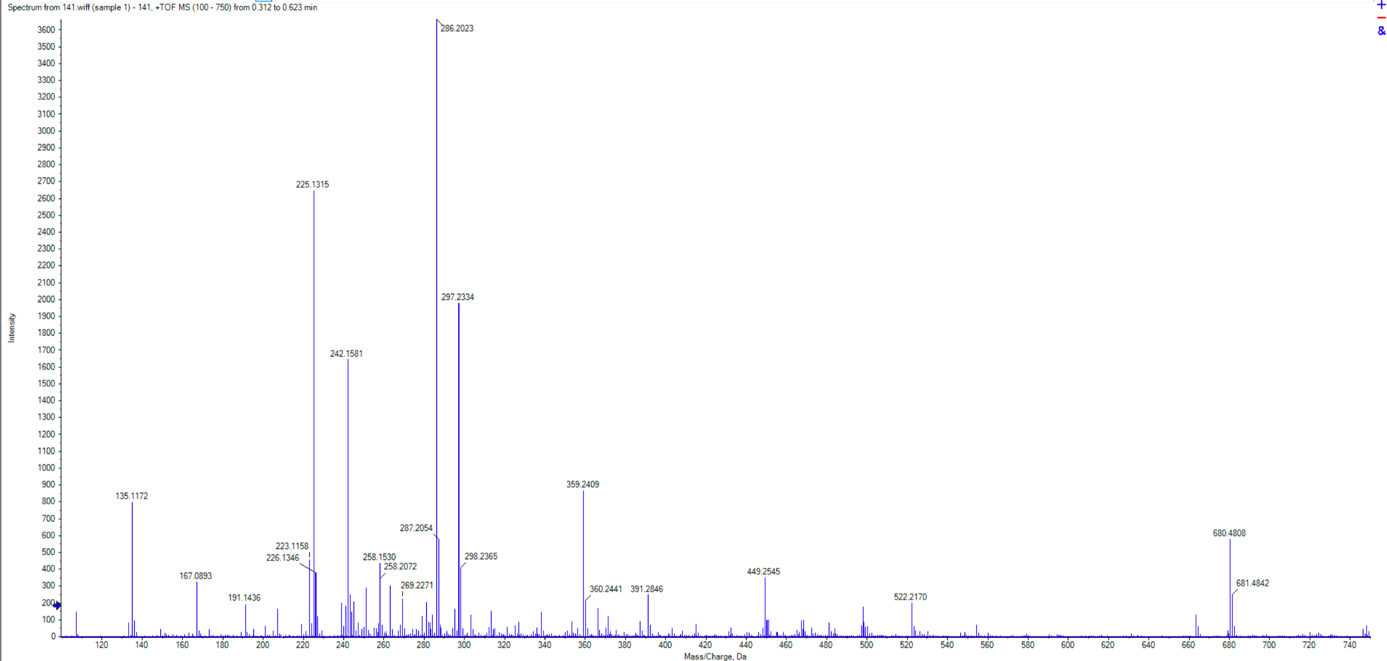
**

Scheme 45. HRMS (ESI) calculated for C_13_H_21_IOS-HI *m/z* = 225.1308 found 225.1315 [M-HI]^+^

Scheme 46. ^1^H NMR spectrum of compound **6a** (300 MHz, CDCl₃)

Scheme 47. ^13^C NMR spectrum of compound **6a** (75 MHz, CDCl₃)


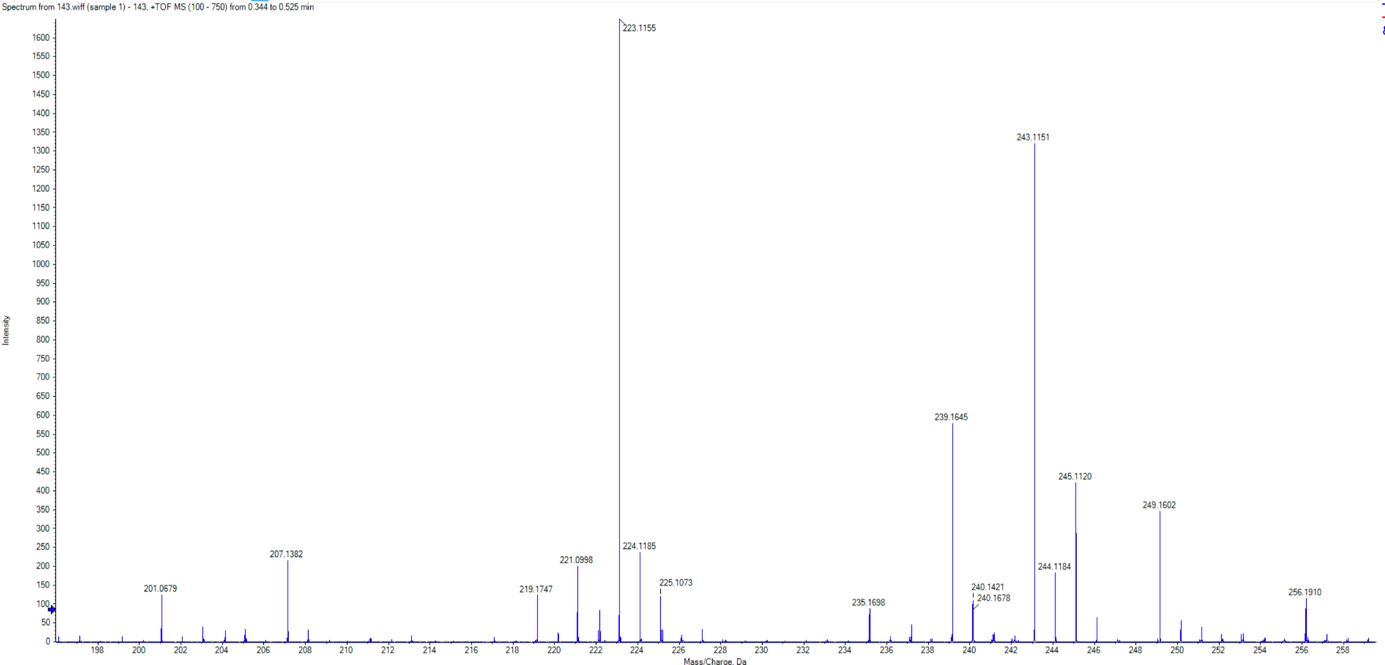


Scheme 48. HRMS (ESI) calculated for C_13_H_19_ClOS-HCl *m/z* = 223.1151 found 223.1155 [M-HCl]^+^

Scheme 49. ^1^H NMR spectrum of compound **6b** (300 MHz, CDCl₃)

Scheme 50. ^13^C NMR spectrum of compound **6b** (75 MHz, CDCl₃)

**
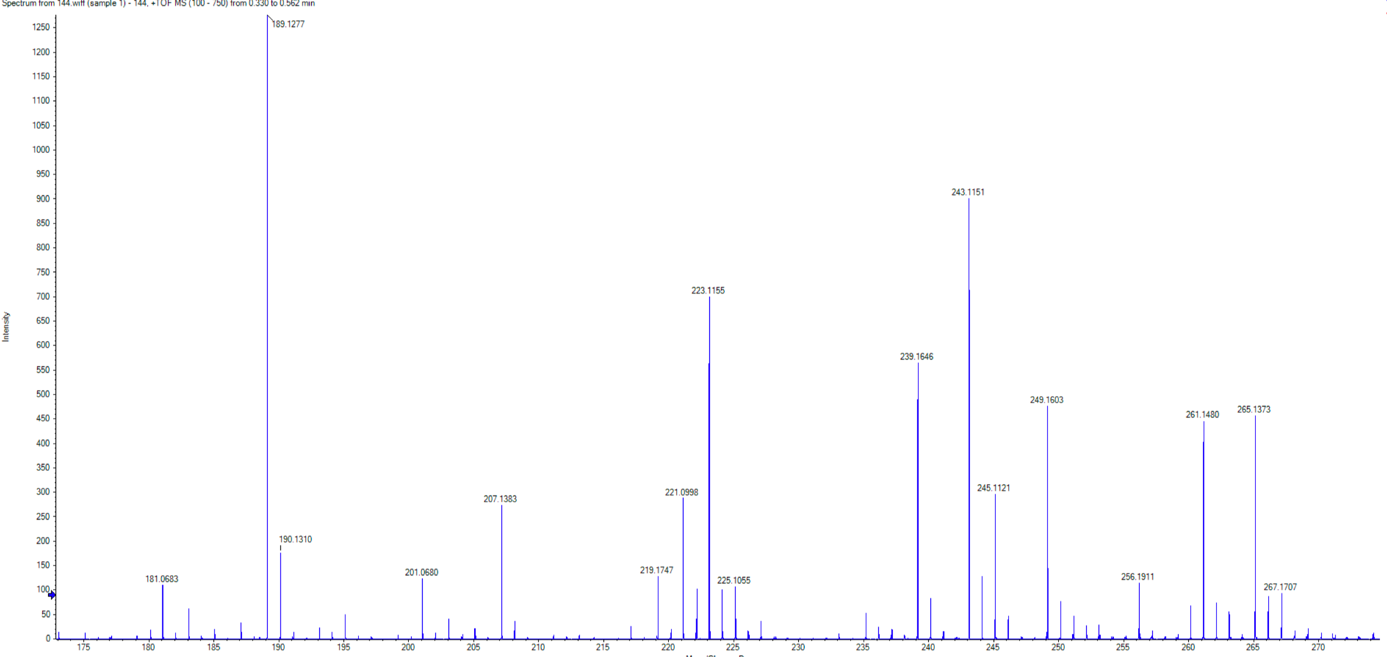
**

Scheme 51. HRMS (ESI) calculated for C_13_H_19_BrOS-HBr *m/z* = 223.1151 found 223.1155 [M-HBr]^+^

Scheme 52. ^1^H NMR spectrum of compound **6c** (300 MHz, CDCl₃)

Scheme 53. ^13^C NMR spectrum of compound **6c** (75 MHz, CDCl₃)

Scheme 54. HRMS (ESI) calculated for C_13_H_19_IOS-HI *m/z* = 223.1151 found 223.1152 [M-HI]^+^

Scheme 55. ^1^H NMR spectrum of compound **7a** (300 MHz, CDCl₃)

Scheme 56. ^13^C NMR spectrum of compound **7a** (75 MHz, CDCl₃)

**
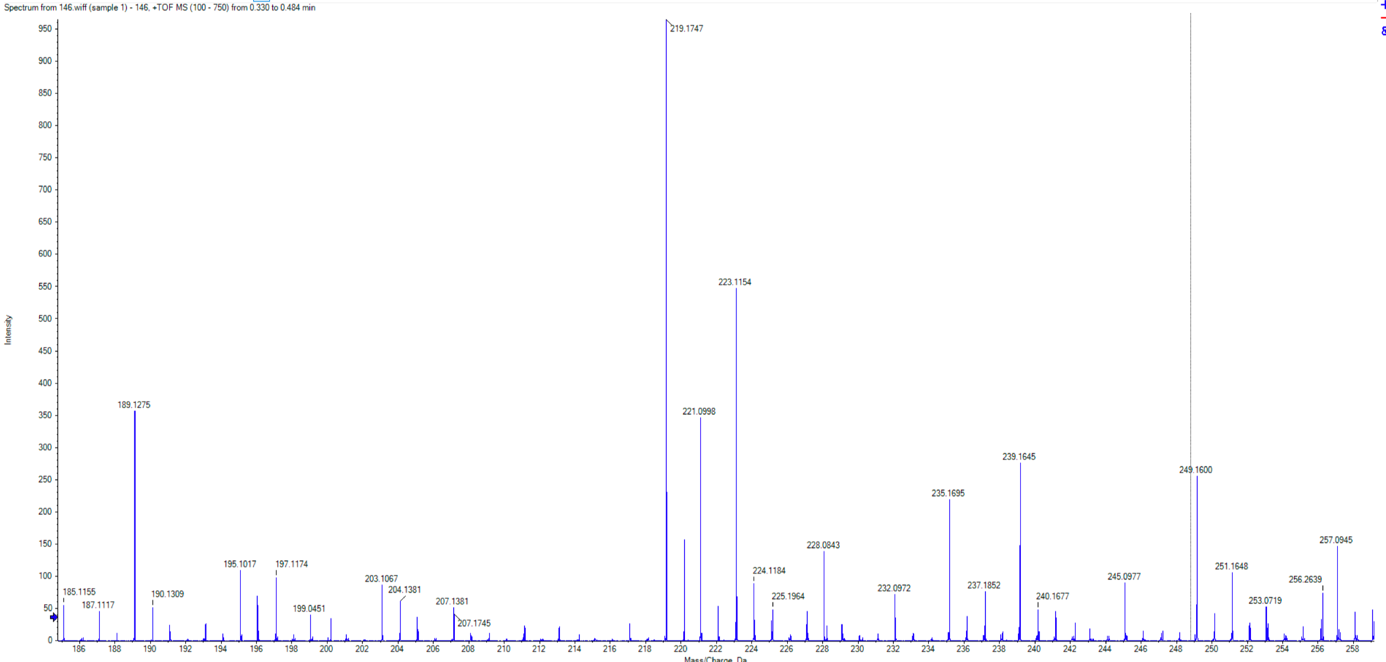
**

Scheme 57. HRMS (ESI) calculated for C_13_H_19_ClOS-HCl *m/z* = 223.1151 found 223.1154 [M-HCl]^+^

Scheme 58. ^1^H NMR spectrum of compound **7b** (300 MHz, CDCl₃)

Scheme 59. ^13^C NMR spectrum of compound **7b** (75 MHz, CDCl₃)


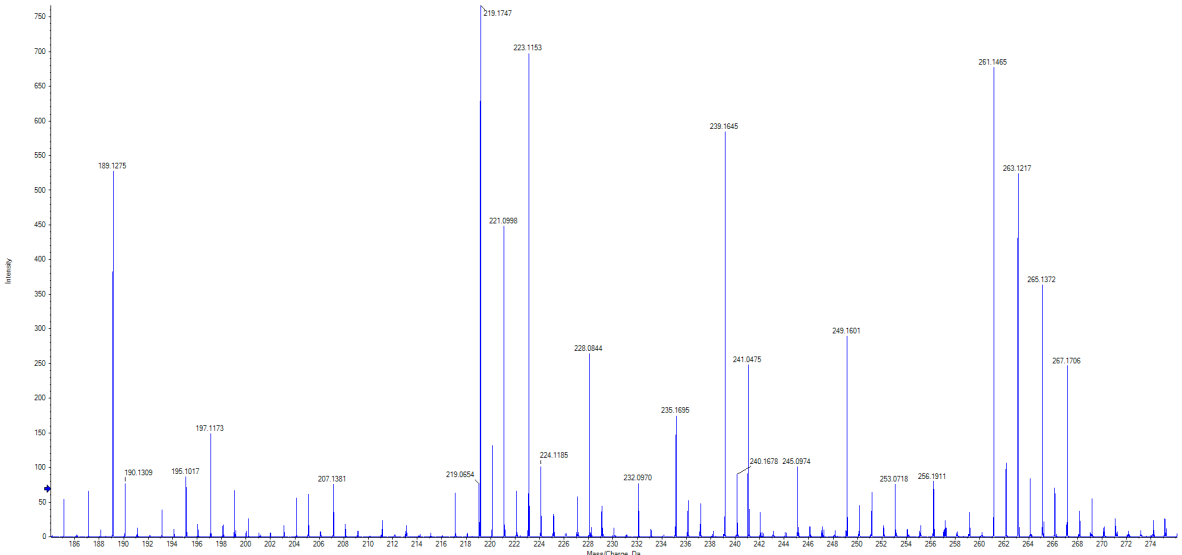


Scheme 60. HRMS (ESI) calculated for C_13_H_19_BrOS-HBr *m/z* = 223.1151 found 223.1153 [M-HBr]^+^.

Scheme 61. ^1^H NMR spectrum of compound **7c** (300 MHz, CDCl₃)

Scheme 62. ^13^C NMR spectrum of compound **7c** (75 MHz, CDCl₃)


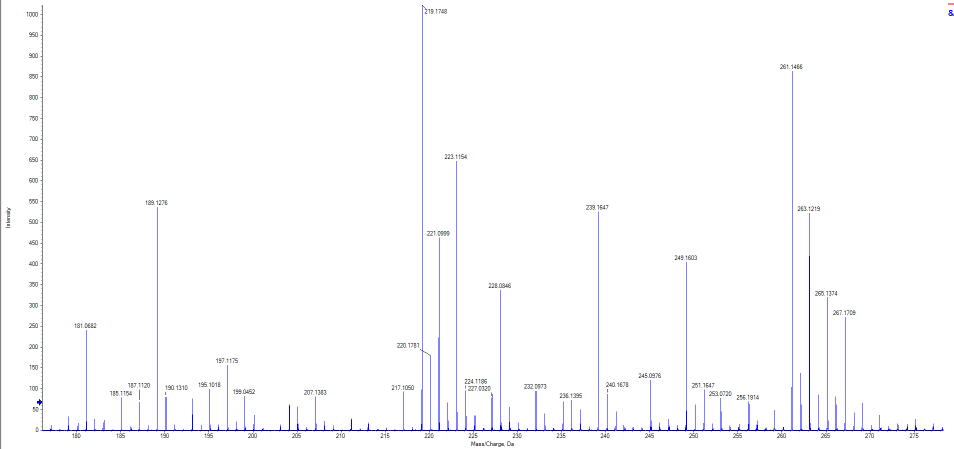


Scheme 63. HRMS (ESI) calculated for C_13_H_19_IOS-HI *m/z* = 223.1151 found 223.1154 [M-HI]^+^

Scheme 64. ^1^H NMR spectrum of compound **8a** (300 MHz, CDCl₃)

Scheme 65. ^13^C NMR spectrum of compound **8a** (75 MHz, CDCl₃)


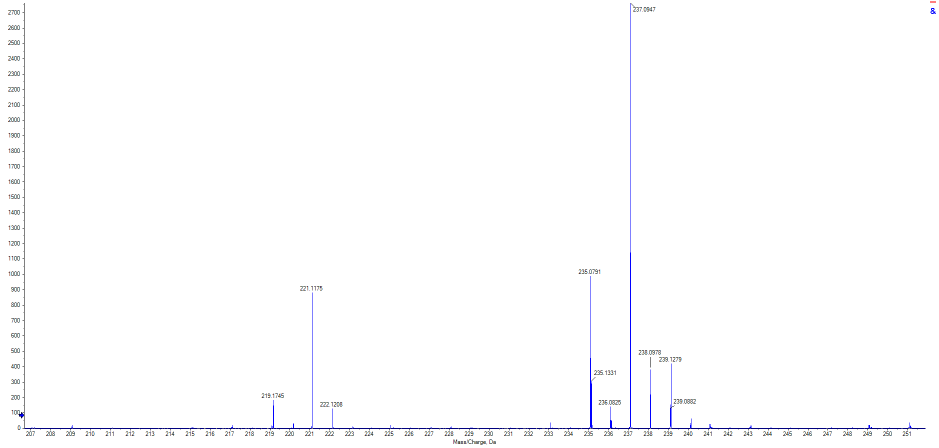


Scheme 66. HRMS (ESI) calculated for C_13_H_17_ClO_2_S-HCl *m/z* = 237.0944 found 237.0947 [M-HCl]^+^

Scheme 67. ^1^H NMR spectrum of compound **8b** (300 MHz, CDCl₃)

Scheme 68. ^13^C NMR spectrum of compound **8b** (75 MHz, CDCl₃)


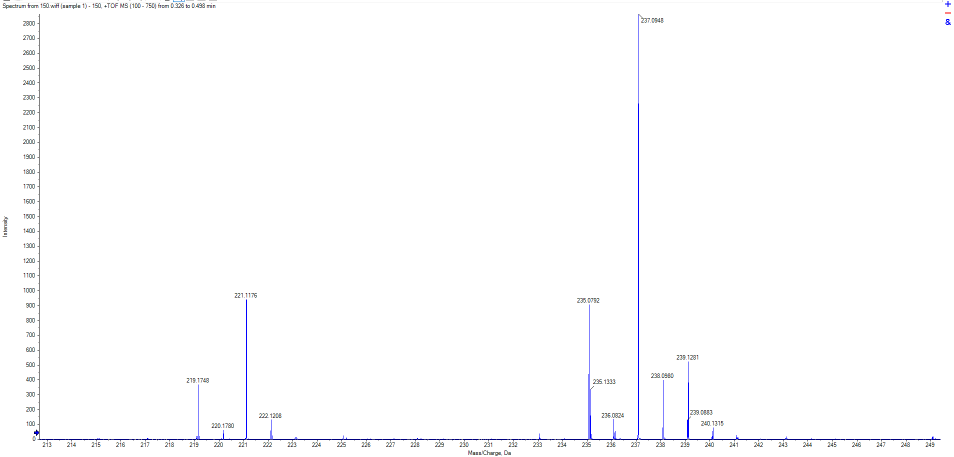


Scheme 69. HRMS (ESI) calculated for C_13_H_17_BrO_2_S-HBr *m/z* = 237.0944 found 237.0948 [M-HBr]^+^

Scheme 70. ^1^H NMR spectrum of compound **8c** (300 MHz, CDCl₃)

Scheme 71. ^13^C NMR spectrum of compound **8c** (75 MHz, CDCl₃)

**
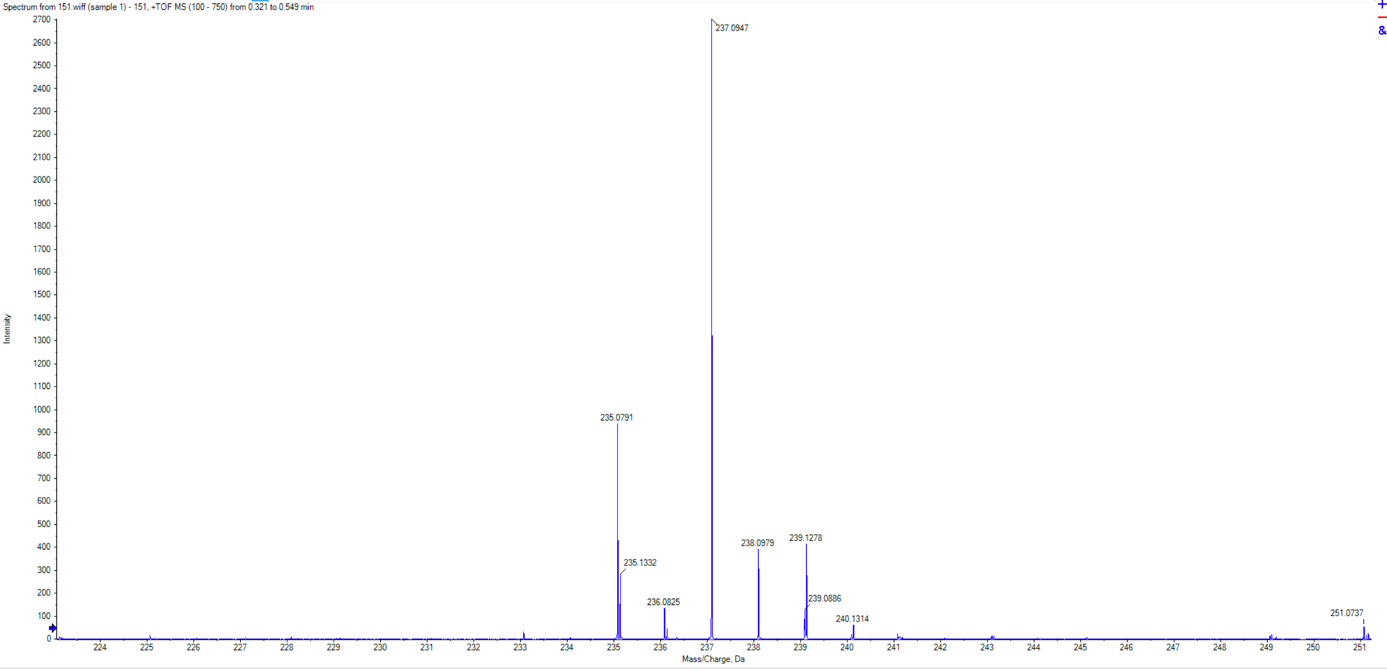
**

Scheme 72. HRMS (ESI) calculated for C_13_H_17_IO_2_S-HI *m/z* = 237.0944 found 237.0947 [M-HI]^+^.
